# Brain Atrophy in Dogs With Meningoencephalitis of Unknown Origin

**DOI:** 10.1111/jvim.70095

**Published:** 2025-06-06

**Authors:** Rita Gonçalves, Gemma Walmsley, Thomas W. Maddox

**Affiliations:** ^1^ Department of Veterinary Science Small Animal Teaching Hospital, University of Liverpool, Leahurst Neston UK; ^2^ Department of Musculoskeletal and Ageing Science Institute of Lifecourse and Medical Sciences, University of Liverpool Liverpool UK

**Keywords:** brain atrophy, canine, MRI, MUO, outcome, prognosis

## Abstract

**Background:**

Information regarding repeat magnetic resonance imaging (MRI) findings in dogs with meningoencephalitis of unknown origin (MUO) is sparse and it is unknown whether brain atrophy occurs.

**Objectives:**

To determine whether brain atrophy occurs in MUO and evaluate if there is an association between atrophy and survival or relapse.

**Animals:**

Twenty‐three dogs diagnosed with MUO that underwent MRI of the brain on two occasions at least six months apart.

**Methods:**

Retrospective study. Interthalamic adhesion thickness to brain height ratio (ITr), lateral ventricle to brain height ratio (LVr), interthalamic adhesion thickness/brain height to lateral ventricle/brain height (ITr/LVr), bicaudate ratio (BCR) and total parenchymal brain volume (TPBV) were measured on both MRI studies and compared.

**Results:**

Thirteen dogs relapsed and four died during the study period. Median time between MRIs was 12 months, and only one imaging study (1/23) was considered normal on the second scan. All MRI variables measured significantly changed between imaging studies, but only higher TPBV was associated with increased survival (OR = 1.59, CI = 1.006–2.51, *p* = 0.047); no variables were found to be associated with relapse. New lesions were identified in six dogs (four of which also showed contrast enhancing lesions), with 5/6 of these dogs subsequently relapsing.

**Conclusions and Clinical Importance:**

Brain atrophy likely occurs in dogs with MUO and is associated with worse outcomes. Clinical relapse might be likely in dogs with new or contrast‐enhancing lesions on repeat MRI, so more aggressive treatment should be considered in these dogs.

**Abbreviations**
BCR: bicaudate ratio
CCD: canine cognitive dysfunction
CI: confidence intervals
CSF: cerebrospinal fluid
FLAIR: fluid attenuating inversion recovery
ICP: intracranial pressure
IQR: interquartile range
ITr: interthalamic adhesion thickness to brain height ratio
ITr/LVr: interthalamic adhesion thickness/brain height to lateral ventricle/brain height
LVr: lateral ventricle to brain height ratio
MRI: magnetic resonance imaging
MS: multiple sclerosis
MUO: meningoencephalitis of unknown origin
NDS: neurodisability scale
NME: necrotizing meningoencephalitis
TNCC: total nucleated cell count
TPBV: total parenchymal brain volume

## 1 Introduction

Meningoencephalitis of unknown origin is the term used to describe a group of suspected immune‐mediated diseases of the central nervous system (CNS) in dogs [1, 2, 3]. It typically includes the subtypes of granulomatous meningoencephalitis (GME), necrotizing meningoencephalitis (NME) and necrotizing leukoencephalitis (NLE) and these can only be distinguished through histopathology. MUO is thought to be one of the most common inflammatory diseases affecting the CNS in dogs [4], with GME contributing to up to 25% of all canine CNS diseases [5].

Magnetic resonance imaging (MRI) remains a crucial step in the diagnosis of MUO in dogs [1, 2]. Findings vary between the different MUO subtypes, and MRI may even be normal [6] but in most cases reveals focal, multifocal, or diffuse T2‐weighted (T2W) and fluid‐attenuating inversion recovery (FLAIR) hyperintensities located in the forebrain, brainstem, or cerebellum [1, 6, 7]. The most commonly affected regions of the brain include the white matter tracts of the corona radiata and corpus callosum, followed by the frontal, sensorimotor, and temporal cortices [8]. Lower T2W lesion burden is associated with longer survival, and higher T1W post‐contrast lesion burden is associated with relapse [8]. Evidence of mass effect, loss of identifiable cerebral sulci, and foramen magnum herniation are associated with increased risk of death [9, 10] but this is not reproducible [8, 11, 12, 13].

Several studies suggest similarities between MUO (particularly the NME form) and multiple sclerosis (MS) in humans [14, 15, 16]. Multiple sclerosis (MS) is a chronic, autoimmune disease of the CNS in humans that is characterized by focal and diffuse inflammation and neurodegeneration leading to axonal loss [17, 18]. MRI lesions in MS are scattered throughout both white matter and gray matter, and abnormal areas of the brain on pathological studies can appear normal on MRI [19]. Brain atrophy, defined as the gradual loss of brain volume, is extensive in MS and its severity is a strong predictor of future cognitive and physical disability, but can be reduced by disease‐modifying drugs [18, 20]. Brain atrophy is reported in a dog with NME, identified through repeat MRI at 21 and 48 months after diagnosis and confirmed on histopathological analysis [21].

The aims of this study were to investigate if brain atrophy is identifiable in dogs with MUO that undergo repeat MR imaging and, if so, whether this is associated with survival and relapse.

## 2 Materials and Methods

The clinical records of dogs diagnosed with suspected MUO and that had undergone at least two brain MRIs with a minimum of a six‐month interval at the Small Animal Teaching Hospital (SATH) of the University of Liverpool were reviewed retrospectively. Ethical approval for use of data was granted by the Ethics Committee of the University of Liverpool (VREC840). A diagnosis of suspected MUO was given when (1) clinical examination was consistent with focal or multifocal intracranial neuroanatomical localization; (2) dogs were older than 6 months of age; (3) multiple, single, or diffuse intra‐axial hyperintensities were identified on T2W MR images; and (4) dogs responded, at least partially, to immunosuppressive treatment. Cerebrospinal fluid analysis was not required for inclusion in this study due to studies showing that it can be normal in a proportion of dogs with MUO [2, 3, 7, 22, 23], but the results were recorded. Dogs were excluded if signs of raised intracranial pressure (namely transtentorial or foramen magnum herniation and effacement of the cerebral sulci) were identified on either the initial or repeat MRI scan because this would suggest considerable brain swelling and a reversible increase in parenchymal volume and decrease in ventricular volume that would complicate comparison between MRI examinations.

The following data were extracted from the clinical records: age, sex, neuter status, breed and duration of the clinical signs and survival information. The findings of the neurological examination recorded on the patient files were used to retrospectively assign the neurodisability scale (NDS) score [24] for each dog (calculated by the same assessor in all dogs) at the time of initial presentation and at the time of repeat MRI.

MRI examinations of the head were performed using a 1.0 T (Siemens Magnetom, Erlangen, Germany) or a 1.5 T (Philips Ingenia CX, Philips Healthcare, Netherlands) scanner; dogs were only included if they underwent the MRI in the same machine. The following sequences were obtained in all dogs: T2‐weighted images (T2W), fluid‐attenuated inversion recovery (FLAIR) and pre‐ and post‐contrast (intravenous injection of 0.1 mmol/kg of gadopentetate dimeglumine) T1‐weighted images (T1W).

**FIGURE 1:**
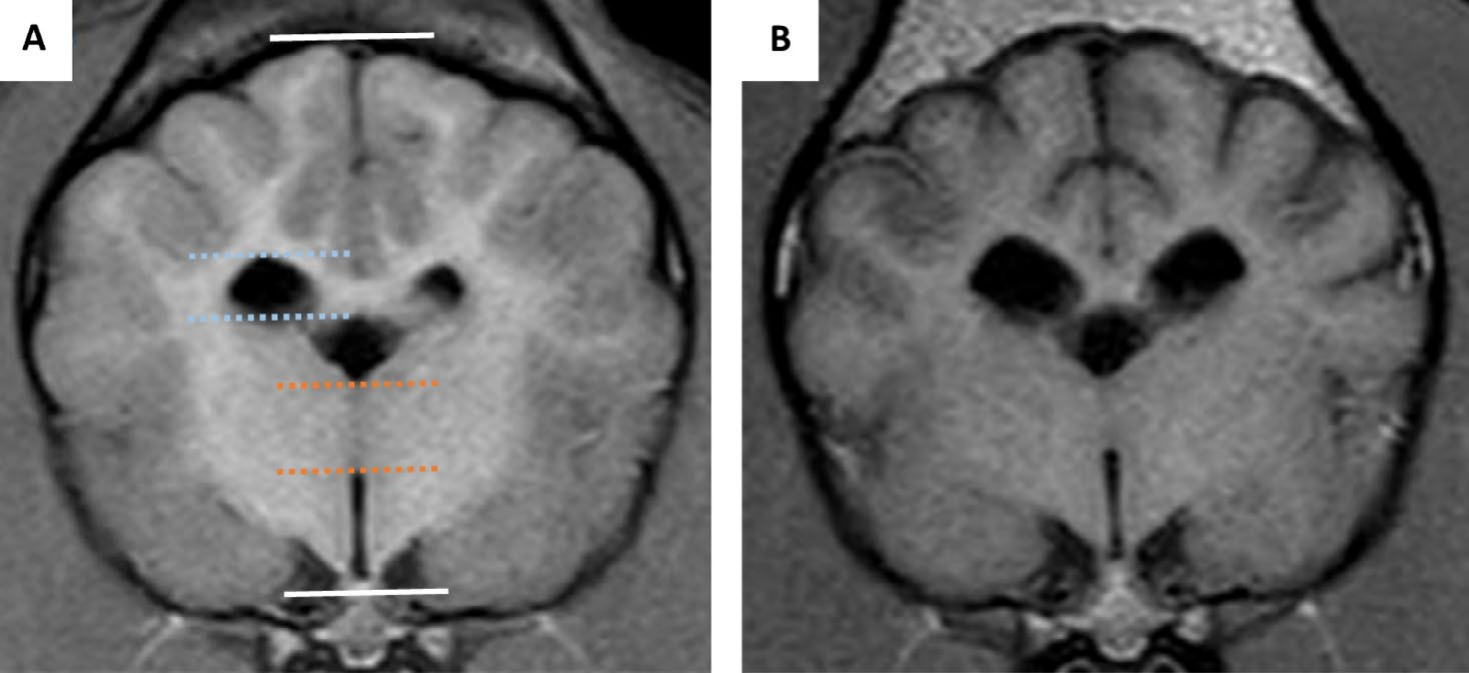
Measurements of the interthalamic (IT) adhesion thickness (dotted orange lines), the maximum ventricular height (dotted blue lines) and maximum brain height at the same level (white lines) in order to calculate the interthalamic adhesion thickness to brain height ratio (ITr), the lateral ventricle to brain height ratio (LVr) and the interthalamic adhesion thickness/brain height to lateral ventricle/brain height (ITr/LVr). (A) Transverse T1WI of a 7‐month‐old French Bulldog with MUO at the level of the IT adhesion and (B) Transverse T1WI of the same dog 28 months later.

**FIGURE 2:**
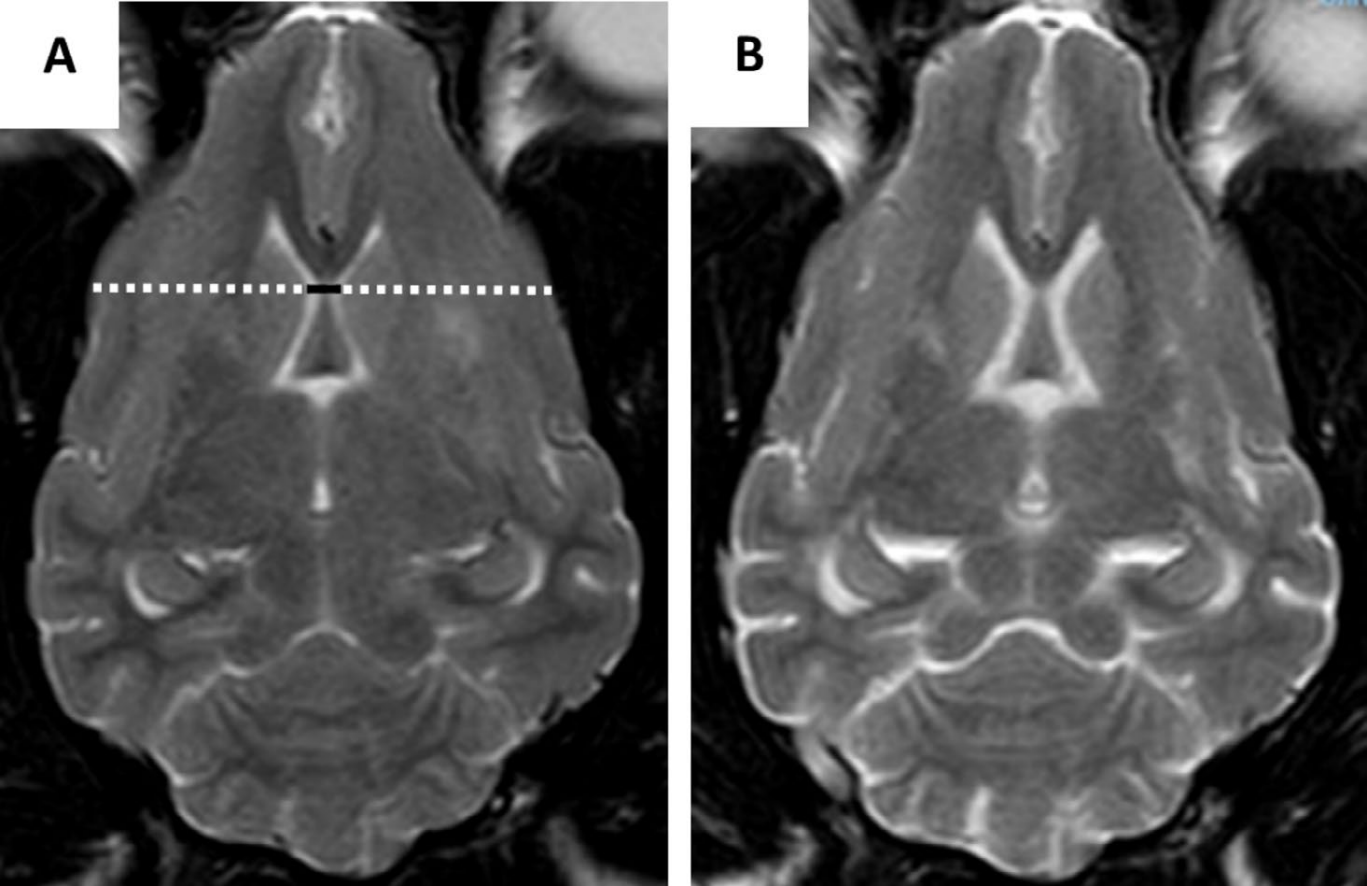
Measurement of the bicaudate ratio (BCR), which represents the minimum intercaudate distance (solid black line) divided by brain width along the same line (dashed white line). (A) Dorsal T2WI of a 10‐year‐old West Highland White terrier with MUO at the level of the caudate nuclei and (B) Dorsal T2WI of the same dog 25 months later.

Several measurements were performed by one observer (RG) who was blinded to the dogs' details using a picture archiving and communication system (PACS) workstation (Carestream Vue PACS Version 11.14, Phillips, Netherlands). Transverse T1W images were used to measure the interthalamic (IT) adhesion thickness, the maximum ventricular height, and maximum brain height at the same level in order to calculate the interthalamic adhesion thickness to brain height ratio (ITr), the lateral ventricle to brain height ratio (LVr) and the interthalamic adhesion thickness/brain height to lateral ventricle/brain height (ITr/LVr) as previously described (Figure 1) [25, 26]. The bicaudate ratio (BCR) was measured in the T2W dorsal plane slice where the heads of the caudate nuclei were most visible and closer to one another. The BCR was the minimum intercaudate distance divided by brain width along the same line (Figure 2). Image processing for volume rendering was performed using graphical software (AMIRA 6.2, Thermo Fisher Scientific, UK). Segmentation techniques used were similar to previous studies in dogs [27, 28]. The total brain volume and the total ventricular volume were measured from individual slices by free‐hand measurements on transverse T1W images. The total parenchymal brain volume (TPBV) was then calculated by subtracting the total ventricular volume from the total brain volume. Ratios were calculated between all measurements performed on the second MRI and the initial one for use in the statistical analysis. The MRI images were also used to assign the dogs to a suspected MUO subtype: GME or necrotising encephalitis (NE—including the subtypes NME and NLE). Dogs were classified as suspected NE when at least one region suggestive of a necrotic/cyst‐like lesion was identified, characterized by being hyperintense on T2W and hypointense on T1W and FLAIR sequences.

Statistical analysis was undertaken using a standard statistical software package (IBM SPSS Statistics, version 29, IBM Corp, Armonk, New York). Continuous data were assessed for normality using the Shapiro–Wilk test. Descriptive statistics are reported for continuous variables using mean (standard deviation) for approximately normally distributed variables, median (interquartile range; IQR) for variables with skewed distributions, and frequencies (with 95% confidence intervals [CI] where appropriate) for categorical variables. Linear regression was performed to identify MRI variables (using the ratios between the repeat and initial MRI for all the different measurements) associated with the time between the two MRIs and also with the NDS score at the time of the second MRI. Binary logistic regression was used to identify MRI measurements associated with survival, relapse, and suspected MUO subtype. Before multivariable analysis, all variables were assessed for correlation using Spearman's rank correlation coefficients. If Spearman's rank correlation coefficient was > 0.8, the most statistically significant or biologically plausible variable was selected for inclusion. Any independent variable demonstrating some association on preliminary univariable analysis (*p* < 0.2) was considered for inclusion in a multivariable model. Multivariable models were constructed with a manual backwards stepwise removal approach; variables with *p* < 0.05 were retained. The Wilcoxon signed‐rank test was used to compare the different MRI measurements between the initial and the second MRI. The Mann–Whitney *U* test was used to investigate differences in CSF total nucleated cell count (TNCC; both at time of diagnosis and at repeat MRI) between dogs that relapsed and those that did not.

## 3 Results

Forty‐seven dogs diagnosed with suspected MUO that underwent repeat MRI were identified. Thirteen dogs were excluded due to the identification of MRI findings consistent with raised intracranial pressure, eight because the MRI interval was less than six months, and three because they were imaged in different MRI scanners on the first and second examinations. A total of 23 dogs were therefore included in the data analysis (Table 1). There were 14 females (12 neutered) and 9 males (six neutered) and the median age at diagnosis was 50 months (IQR 25–84). Breeds affected included the French bulldog (*n* = 5), crossbreeds (*n* = 4), Chihuahua (*n* = 3), Maltese terrier (*n* = 2) and one each of the following breeds: Bedlington terrier, Bernese Mountain dog, Cavalier King Charles spaniel, Cocker spaniel, Golden retriever, Jack Russell terrier, miniature Pinscher, Pug, and West Highland white terrier. Based on the MRI characteristics, 14 dogs had suspected GME and nine suspected NE. All dogs received immunosuppressive doses of dexamethasone at the time of diagnosis alongside cytosine arabinoside in 20 dogs (subcutaneous injection in 6 dogs and constant rate infusion in 14). Immunomodulatory drugs used long term varied substantially between dogs in terms of dose and length of treatment but included prednisolone in all dogs, cytosine arabinoside administration every 3–8 weeks (8), cyclosporine (7) and leflunomide (6).

**TABLE 1:** Clinical and magnetic resonance imaging findings of the 23 dogs diagnosed with suspected MUO at the different assessment times.

	Initial MRI	Repeat MRI
Number of lesions		
Focal	7/23 (30%, [95% CI 0.13–0.53])	9/23 (39%, [95% CI 0.2–0.61])
Multifocal	16/23 (70%, [95% CI 0.47–0.87])	14/23 (61%, [95% CI 0.39–0.8])
Lesion(s) location		
Forebrain	19/23 (83%, [95% CI 0.61–0.95])	19/23 (83%, [95% CI 0.61–0.95])
Brainstem	15 (65.2%, [0.46–0.85])	14/23 (61%, [95% CI 0.39–0.8])
Cerebellum	2 (8.7%, [95% CI 0.0–0.28])	0/23
Spinal cord	2 (8.7%, [95% CI 0.0–0.28])	1/23 (4%, [95% CI 0.01–0.22])
Optic nerves	0/23	1/23 (4%, [95% CI 0.01–0.22])
Contrast enhancement	21/23 (91%, [95% CI 0.72–0.99])	4/23 (17%, [95% CI 0.05–0.39])
Median (IQR) NDS score	4 (2–6)	0 (0–3)
Median (IQR) CSF TNCC	12 (4–60)	3 (1–13)
Median (IQR) CSF protein	0.3 (0.23–0.92)	0.32 (0.22–0.39)

Abbreviations: CI, confidence interval; CSF, cerebrospinal fluid; IQR, interquartile range; MRI, magnetic resonance imaging; NDS, neurodisability scale; TNCC, total nucleated cell count.

Median time interval between MRIs was 12 months (IQR 7–25) and median follow up time was 30 months (IQR 20–40). The second MRI was performed for monitoring progress in 16 cases, of which 11 were neurologically normal on repeat examination and five had responded to treatment but residual deficits remained. In five cases, the repeat MRI was performed due to suspected deterioration and in the remaining two cases because of slow progressive disease.

**FIGURE 3:**
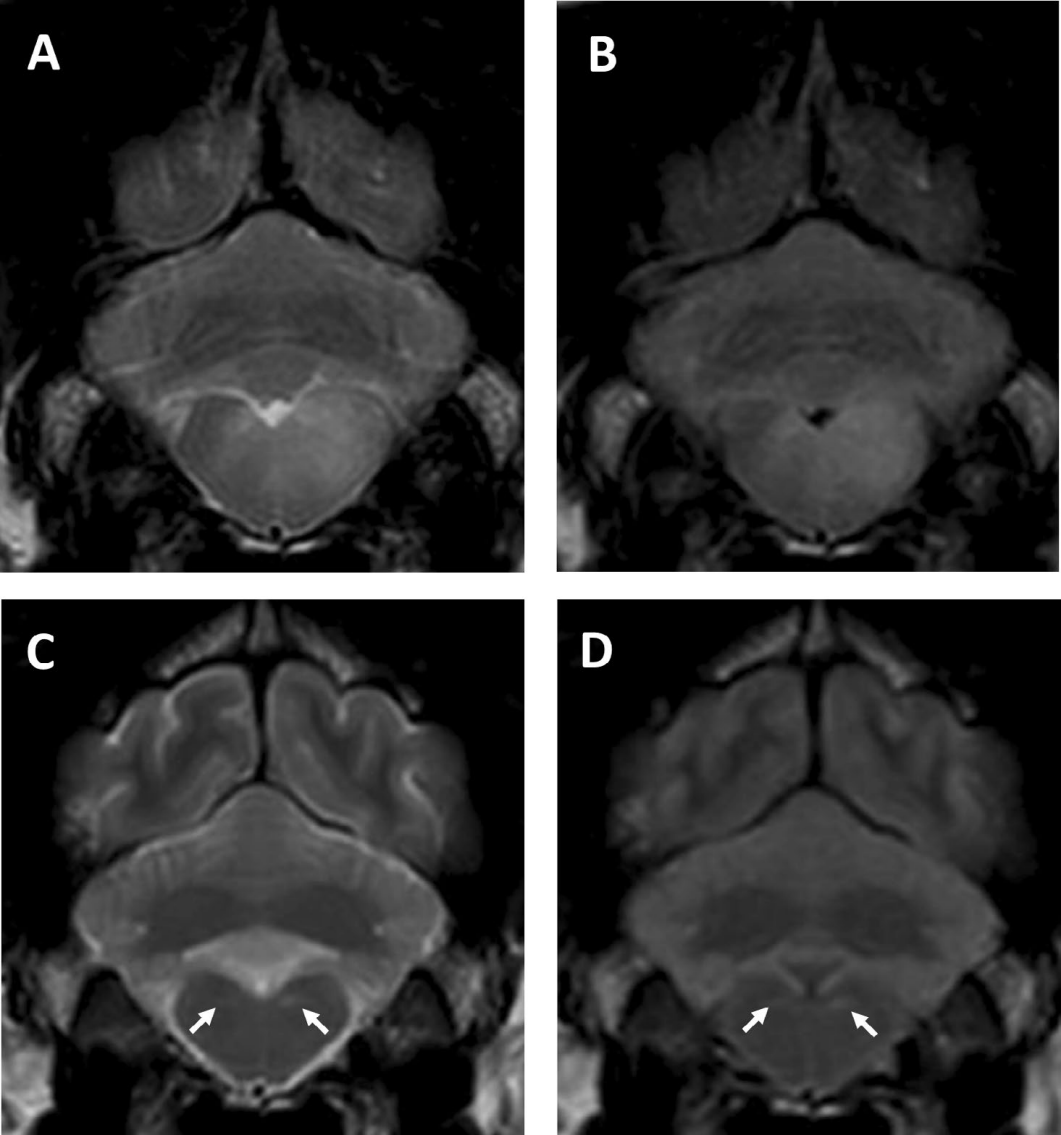
Magnetic resonance imaging (MRI) of a 5‐year‐old crossbreed with MUO on initial MRI (A–B) and 12‐months later (C–D); (A) and (C) are transverse T2‐weighted (T2W) images and (B) and (D) are transverse fluid attenuated inversion recovery (FLAIR) images. In the initial MRI, a large T2W and FLAIR hyperintense lesion can be seen at the level of the medulla oblongata but on repeat MRI, only two small residual hyperintense lesions remain. This dog was neurologically normal at the time of repeat MRI.

**FIGURE 4:**
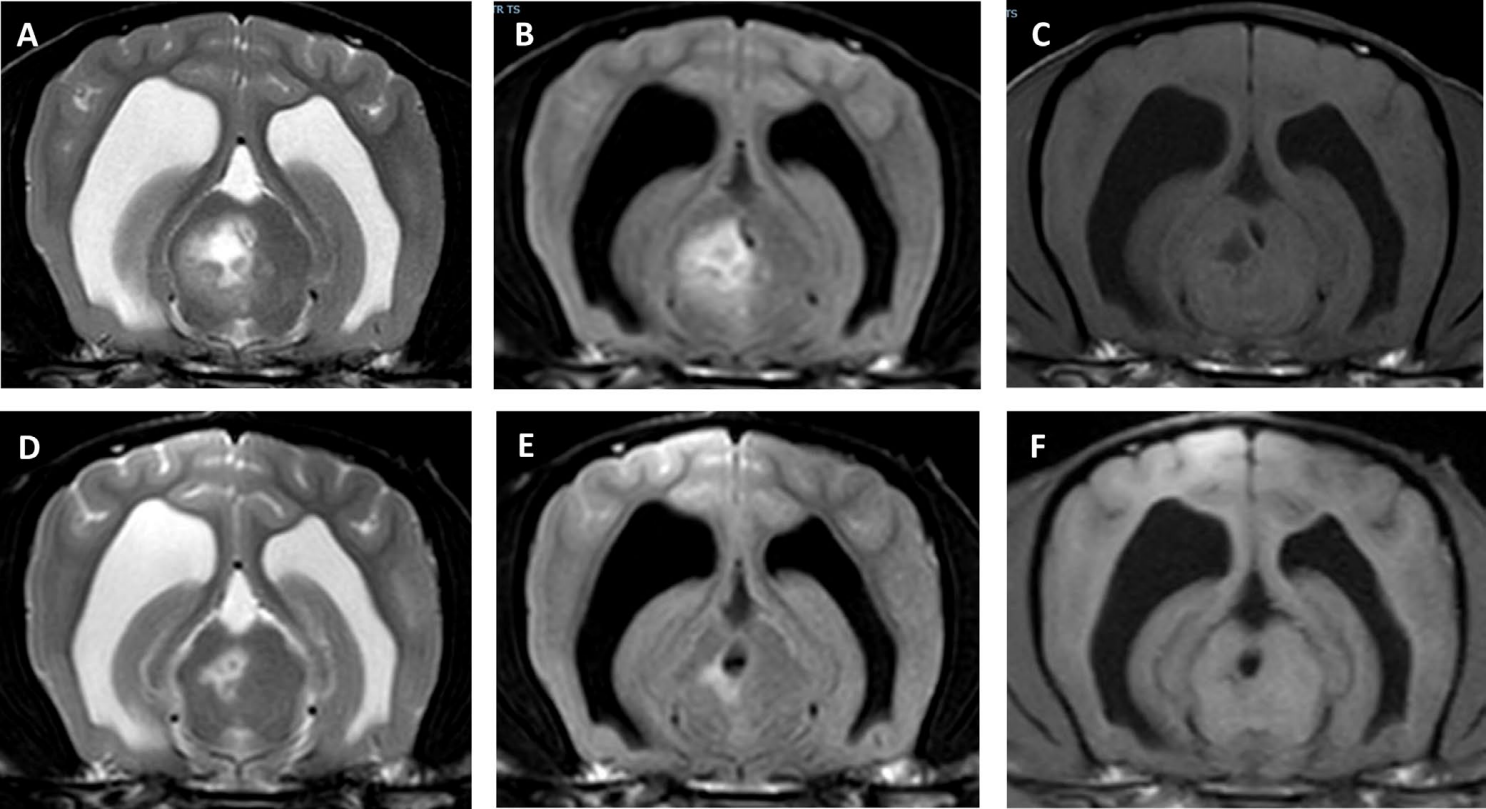
Magnetic resonance imaging (MRI) of a 4‐year‐old Chihuahua with MUO on initial MRI (A–C) and 9‐months later (D–F); A and D are transverse T2‐weighted (T2W) images, (B) and (E) are transverse fluid attenuated inversion recovery (FLAIR) images and (C) and (E) are T1‐weighted (T1W) images. In both MRIs, the same lesion appears hyperintense on T2W and FLAIR and hypointense on T1W images but the size is smaller on the second MRI. This dog was neurologically normal at the time of repeat MRI.

**FIGURE 5:**
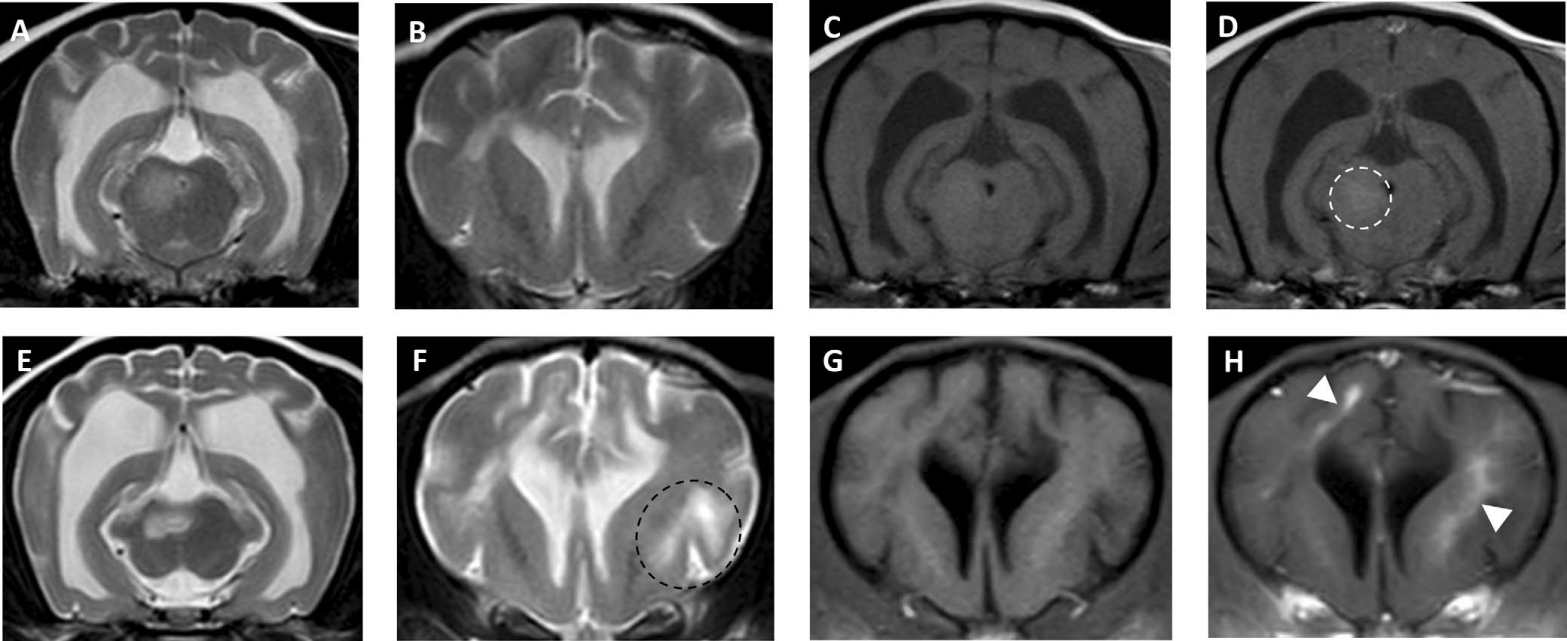
Magnetic resonance imaging of a 3‐year‐old crossbreed with MUO that showed clinical improvement but remained with permanent neurological deficits at the time of initial diagnosis of MUO (A–D) and 12‐months later (E–F). Images (A–B) and (E–F) are transverse T2‐weighted (T2W) images, showing enlargement of the lateral ventricles and sulci, suggesting parenchymal atrophy and also development of a new lesion in the frontal cortex (black dotted circle) not seen in the initial MRI. Images (C) and (G) are transverse T1‐weighted (T1W) images and (D) and (H) are transverse T1W post‐contrast images showing the only mildly contrast enhancing lesion in the initial MRI (dotted white circle) and the new areas of contrast enhancement in the second MRI (white arrowheads).

At the time of the second MRI, only one imaging study was considered normal (Table 2). In some dogs, the lesions almost resolved but subtle (Figure 3) or smaller but still prominent changes (Figure 4) in the areas previously affected could still be detected on T2WI and FLAIR. Of the five dogs with suspected relapse, two had new lesions attributed to MUO (one dog developed new signs of optic neuritis and the other had new signs localizing to the spinal cord and was diagnosed with meningomyelitis which was not evident at initial presentation), two had a presumptive diagnosis of idiopathic vestibular disease (based on acute peripheral vestibular signs and clinical improvement with no changes to the treatment), and one had an intervertebral disc extrusion in the cervical region. The two dogs with slow progressive clinical signs that showed only minor response to treatment over a one‐year period in one dog and four years in the other also showed new lesions as well as parenchymal contrast enhancement on repeat MRI (Figure 5). A further two of the 16 dogs that were scanned for monitoring purposes also showed new lesions that had not been identified in the initial MRI—both had shown clinical improvement with treatment but neurological deficits remained.

**TABLE 2:** Findings at the time of repeat magnetic resonance imaging (MRI) of the brain of the 23 dogs diagnosed with suspected MUO.

Repeat MRI (*n* = 23)	Hypointense on T1‐weighted images	Contrast enhancement	Clinical relapse
Normal *n* = 1	0/1	0/1	0/1
Subtle lesion *n* = 4	2/4	0/4	1/4
Fewer and smaller lesions *n* = 7	3/7	0/7	5/7
Same number lesions but smaller size *n* = 4	4/4	0/4	1/4
No significant changes *n* = 1	1/1	0/1	1/1
Smaller lesions but new areas affected *n* = 4	2/4	2/4	3/4
More and larger lesions *n* = 2	2/2	2/2	2/2

*Note:* All lesions identified were hyperintense on T2‐weighted and fluid‐attenuated inversion recovery (FLAIR) images.

Hence, a total of six dogs showed new lesions that had not been identified in the initial MRI. Four of these dogs showed mononuclear pleocytosis on CSF analysis, one showed albuminocytological dissociation, and in one dog, CSF was not collected at the time of the second MRI. In four dogs, contrast‐enhancing lesions were observed on the repeat MRI; this included the two dogs showing relapse (optic neuritis and meningomyelitis) and the two dogs with progressive disease.

Thirteen dogs were suspected of clinical relapse within a median time of 18 months (IQR 13–21) and four dogs died during the follow‐up period (these dogs had never recovered completely and had clinical relapses); post‐mortem was not available for any of these dogs, as they were euthanized at their referring veterinary practices.

At the time of diagnosis, CSF analysis was performed in 19 dogs (iatrogenic blood contamination precluded analysis in 2 dogs and sampling was unsuccessful in two Chihuahuas with Chiari‐like malformation) and showed mononuclear pleocytosis in 17 dogs, albuminocytological dissociation in one dog, and it was normal in one dog (both latter dogs were French bulldogs). Cerebrospinal fluid analysis was repeated alongside the MRI in 18 dogs and showed mononuclear pleocytosis in eight dogs, albuminocytological dissociation in four dogs, and the remaining six dogs had normal CSF. There was no association between the CSF TNCC either at the time of diagnosis (*p* = 0.31) or at the time of repeat MRI (*p* = 0.62) and occurrence of relapse.

The Wilcoxon signed‐rank test showed that all MRI variables measured changed significantly between imaging studies (Table 3). On univariable linear regression, ITr, LVr, and BCR showed some correlation (*p* < 0.2) with both the time interval between MRIs and with the NDS score at the time of the second MRI. However, on multivariable analysis, only lower ITr remained correlated with the time interval between MRIs (*R* ^2^ = 0.25, *p* = 0.016) and higher LVr correlated with the NDS score (*R* ^2^ = 0.31, *p* = 0.01). On univariable binary logistic regression, TPBV, LVr, and ITr/LVr showed some association with both survival and suspected MUO subtype (*p* < 0.2); no associations were found between any of the MRI variables and relapse. On multivariable analysis, only higher TPBV remained associated with increased survival (OR = 1.59, 95% CI = 1.006–2.51, *p* = 0.047); no variables remained significantly associated with suspected MUO subtype.

**TABLE 3:** Comparison of the magnetic resonance imaging measurements from the initial and repeat MRI using the Wilcoxon signed‐rank test in 23 dogs diagnosed with suspected MUO.

	Initial MRI	Repeat MRI	*p* value and *Z* value
Median (IQR) TPBV (mm^3^)	74 841 (60390–85 756)	73 020 (60560–78 066)	*p* < 0.001 *Z* = −3.59
Median (IQR) ITr	17% (15–17.6)	14% (12.6–16.1)	*p* = 0.003 *Z* = −2.99
Median (IQR) LVr	16% (12.6–21.2)	20% (14.4–24.4)	*p* = 0.004 *Z* = −2.91
Median (IQR) ITr/LVr	112% (69.5–134.7)	72% (50–113.2)	*p* = 0.004 *Z* = −2.86
Median (IQR) BCR	11% (9.9–13.9)	13% (12–14.8)	*p* = 0.04 *Z* = −2.05

Abbreviations: BCR, bicaudate ratio; IQR, interquartile range; ITr, interthalamic adhesion thickness to brain height ratio; ITr/LVr, interthalamic adhesion thickness/brain height to lateral ventricle/brain height ratio; LVr, lateral ventricle to brain height ratio; MRI, magnetic resonance imaging; TBPV, total brain parenchymal volume.

## 4 Discussion

Our study showed that brain atrophy is likely a feature of MUO in dogs and it is associated with a worse outcome. Lesions identified on MRI at the time of diagnosis are unlikely to completely resolve within the first 6–12 months after diagnosis and new lesions can develop in dogs with no perceived clinical deterioration. The relapse rate was similar to previous reports but was not associated with any MRI measurement or with the CSF TNCC.

This study revealed that all MRI measurements undertaken significantly changed over time, suggesting that brain atrophy occurs in dogs with MUO. Several mechanisms are expected to affect brain volume in dogs with MUO, and factors likely contributing to brain atrophy include axonal loss, gliosis, demyelination, anti‐inflammatory medications, and normal aging [29]. It also showed that reduction in TBPV was associated with decreased survival. Brain atrophy is quite severe in MS, reported at a rate of 0.5%–1.35% per year, which is greater than what is expected with normal aging [18]. It arises early in the course of disease, with cortical thinning detectable at clinical onset [30] and even before disease becomes symptomatic [31], and accelerates with disease progression [32]. Neuropathology studies have shown that MRI‐measured cortical volume changes in chronic MS patients were predominantly explained by neuronal density and axonal lesions, and there was a less important role for myelin loss [33]. Importantly, brain atrophy is significantly associated with physical and cognitive disability and is a strong predictor of future neurological impairment [18], with short‐term changes (over the first year) predicting clinical status [17]. Our findings suggest a similar association between brain atrophy and outcome, although some of the changes in TBPV might be attributed to normal aging between the MRI studies. The median age at the time of diagnosis in these dogs was four years, and the time interval between the studies was generally around one year, so marked changes would not be expected in this population during that time period. The use of matched age healthy dog controls would be necessary to fully evaluate the effect of aging in these measurements, but this could not be ethically justified in view of the need to anesthetize dogs for the MRI procedures. Interestingly, no association was found between the different MRI measurements and the suspected clinical MUO subtype. We would have hypothesized that atrophy would be most pronounced in NE cases, but this was not the case in our study group. This might be due to the small number of cases included, inherent difficulties with subclassification based on imaging findings alone, the need for a longer interval between the MRI studies for this to develop, or it might be that total parenchymal brain atrophy is similar between GME and NE.

Some of the measurements used in this study, namely the ITr and ITr/LVr, are shown to be lower in dogs with canine cognitive dysfunction (CCD) compared to young and aging dogs with no signs of CCD [25] and the IT adhesion size itself shown to be significantly different between dogs with and without CCD [34, 35, 36]. These changes are not specific for CCD but considered to be indicative of a generalized neurodegenerative process [34, 37, 38, 39]. The ITr difference was the only variable significantly associated with the time interval between MRIs, with IT adhesion reductions thought to represent enlargement of the third ventricle as a consequence of atrophy of the thalami and probably the cortex as well [34]. All other variables significantly changed between the MRIs but were not correlated with the time interval between them. Increases in the LVr were associated with a higher NDS score at the time of the second MRI, suggesting these dogs had more severe residual deficits. Similar findings have been reported in patients with MS, where progressive ventricular enlargement is seen over time and associated with disability status [40].

Only one of the repeat MRI examinations was considered to be completely normal. These findings contradict a previous study where repeat MRI at 3‐months showed complete resolution of the baseline MRI abnormalities in 7/17 dogs [9]. This discrepancy might be explained by the fact that the previous study used a low‐field MRI, so it is possible that more subtle lesions were not visible. In humans with MS, it is well established that disease activity measured by MRI is more sensitive than clinical disease activity for predicting relapse [41]. For this reason, regularly repeated MRI examinations (every 6–12 months) are used as a monitoring tool, and the most reliable variables for subclinical disease are the detection of contrast enhancing lesions and the detection of active (new or enlarging) T2W lesions [42]. The commonly termed “black holes” (T1W hypointense lesions) are thought to reflect persistent damage and correlate better with persistent disability [41]. Also, in six cases, new lesions in areas that had not been seen on the initial MRI were identified on the repeat scan despite receiving immunomodulatory drugs, and all but one of these dogs subsequently relapsed. Progression of lesion load in the first year in patients with MS is correlated with earlier progression of impairment and higher risk of therapeutic failure [42] as was seen in the dogs in this study.

The BCr was evaluated in this study cohort as it is easy to calculate on conventional dorsal MR images, is a useful measure that reflects subcortical atrophy and correlates with cognitive dysfunction in patients with MS [43]. Although it increased significantly during the study period, the change in the ratio was not significantly associated with survival or relapse in this study group. Other two‐dimensional MRI measurements commonly used in patients with MS show to be useful markers of brain atrophy that are associated with outcome include the corpus callosum index (CCI) [44, 45] and the third ventricle width (3VW) [46, 47] and might show similar usefulness in dogs with MUO. These were not measured in the current study as they require 3D images to be available in order to allow complete visualization of these structures and such images were only available for a small number of the dogs in our study.

The relapse rate in our study group was 56%, which is similar to that previously reported in dogs with MUO, although the median time to relapse (18 months) was longer than the reported 7–10 months [9, 24, 48, 49]. It is likely that our study cohort is somewhat biased toward milder MUO phenotypes, as we excluded dogs with raised ICP on the MR studies, which was essential in order to investigate possible brain atrophy. In dogs with raised ICP, the TBPV would have been artificially increased and therefore an overestimate of the degree of brain atrophy after treatment would be expected. Therefore, it is likely some of our results might not be valid for all dogs with MUO, but they do offer important information regarding the progress of this debilitating disease.

There were several limitations in this study. An important limitation is the lack of healthy control dogs with repeated imaging studies at similar times to evaluate the effect of aging on the MRI variables evaluated. Unfortunately, MRI in animals requires general anesthesia, making these procedures invasive and expensive; therefore, precluding the use of a control population in the study. The retrospective nature of the study means that the interval between imaging studies was not standardized, and preferably, longer intervals would have been used. The treatments received varied, and this likely will have an impact on survival and relapse in the dogs. Unfortunately, prospective, randomized clinical trials assessing treatment efficacy are not available, and this results in a large variation of treatment protocols used. Lastly, larger numbers of dogs and sequential MRI monitoring over several years will be necessary to fully evaluate these preliminary findings.

In conclusion, the interpretation of repeat MRI for monitoring of dogs diagnosed with MUO remains challenging, as complete resolution of the initially identified lesions is not common. Brain atrophy likely occurs in dogs with MUO and is associated with a worse outcome. The identification of lesions in areas of the brain previously unaffected and the presence of contrast‐enhancing lesions should raise concern for subsequent treatment failure.

## Disclosure

Authors declare no off‐label use of antimicrobials.

## Ethics Statement

Approved by the Ethics Committee of the University of Liverpool (VREC840). Authors declare human ethics approval was not needed.

## Conflicts of Interest

The authors declare no conflicts of interest.
